# Utilisation of sphericity indices in the assessment of left ventricular shape and function after surgical ventricular restoration in patients recovered from anterior myocardial infarction

**DOI:** 10.1186/1749-8090-8-S1-O175

**Published:** 2013-09-11

**Authors:** P Dabic, S Borovic, S Gradinac, AN Neskovic

**Affiliations:** 1 Department of Cardiac Surgery Dedinje Cardiovascular Institute, Belgrade, Serbia; 2 Internal Medicine Clinic, University Clinical Hospital Center Zemun-Belgrade, Serbia

## Background

Surgical ventricular restoration (SVR) after anterior myocardial infarction with post infarction aneurysm, significantly improves left ventricular (LV) performance. To describe mid-term postoperative changes of shape and performance of the newly created LV and mitral valve function through the classical (SI) and apical (ASI) left ventricular sphericity index, and determinants of mitral regurgitation.

## Methods

A total of 26 consecutive patients underwent SVR between November 2002 and January 2004 at our institution. Transthoracic echocardiogram (TTE) was performed before, 3.1 months).±immediately postoperatively and during follow up (follow up of 17 SVR was performed with the use of Mannequin Endoventricular Shaper filled with 50-60 ml/m2 of saline to size and re-shape residual cavity and to create a new apex. Besides the common TTE parameters of LV morphology and function, we evaluated systolic and diastolic changes in SI, ASI and degree of MR.

## Results

Following SVR, there was a statistically significant decrease in NYHA functional 58,35±0,00098). EDV, ESV and EF improved (176,53<0.51 p±0,60 to 1.53±class (2.45 8,68 to±0.01; 35,1<38,83 p±41,09 to 94,50±0.02; 111,09<46,94 ml p±to 154,02 0,91 to±0.009), without significant change in E/A and DT (1,17<9.39% p±41,06 75,64 msec. NS) but with improvement in IMP±69,99 to 209,64±0,74 NS; 207,54±1,29 0.047).There was a statistically significant<0,15 p±0,21 to 0,501±(0,62 0.004;<0,11p±0,12 to 0,53±improvement in systolic and diastolic SI (0,46 0.01;<0,14 p±0,18 to 0,71±0.00001) and ASI (0,81<0,09 p±0,10 to 0,62±0,52 0.0001) as in the decrease in degree of MR severity<0,13 p±0,10 to 0,67±0,76 0.018).

## Conclusion

SI<0,49 p±1,13 to 0,17±immediately postoperatively (0,76 and ASI enable simple and clinically useful definition of complex morphological and functional changes of the distal part of the left ventricle, as preoperatively, and postoperatively, after the SVR and formation of the new LV apex.

